# Challenges in diagnosis of pulmonary mucoepidermoid carcinoma: A case report

**DOI:** 10.1097/MD.0000000000017684

**Published:** 2019-11-01

**Authors:** Xiangxiang Zhou, Min Zhang, Xingyan Yan, Yulan Zhong, Siyun Li, Jixiang Liu, Linfeng Peng, Xin Gan

**Affiliations:** a Department of Respiratory and Critical Care Medicine, Jiangxi provincial Chest Hospital, Nanchang, Jiangxi, China; b Department of Cardiology, Union Hospital, Tongji Medical College, Huazhong University of Science and Technology. Wuhan, China; c Department of Infectious Disease, The First Affiliated Hospital of Shantou University Medical College, Shantou; d Clinical Medicine, Medical College of Nanchang University, Nanchang, China.

**Keywords:** lung neoplasm, pathological histlology, pulmonary mucoepidermoid carcinoma, ThinPrep cytology test, treatment

## Abstract

**Rationale:**

Pulmonary mucoepidermoid carcinomas (PMECs) of the lung are rare malignant tumors. Despite progresses in examinations, the tumor represents a diagnostic challenge for pathologists and clinical physicians. Here, we present a patient who was eventually diagnosed with PMEC by the bronchoscopic examinations conducted three times.

**Patient Concerns:**

We present the case of a 41-year-old female who was initially diagnosed with pulmonary pleomorphic adenoma (PPA) with a 68 × 82 mm mass and nodules in her lung and eventually diagnosed with PMEC.

**Diagnoses:**

Based on histopathology, immunohistology, and imaging studies, the patient was diagnosed with PMEC (pT4N2M1).

**Interventions:**

The patient received first-line systemic chemotherapy regime (gemcitabine combined with carboplatin).

**Outcomes:**

The patient received 2 cycles of chemotherapy. Based on the response evaluation criteria in solid tumor, she achieved partial response, and the mass was distinctly decreased from 68 × 22 mm to 41 × 17 mm.

**Lessons:**

This case presents a rare PMEC overlapping with PPA, based on histological findings, suggesting that besides imaging studies and laboratory examinations, multiple biopsies and ThinPrep cytology tests are necessary to obtain an accurate diagnosis. The patient showed positive response to chemotherapy.

## 1 Introduction

Pulmonary mucoepidermoid carcinoma (PMEC) is a rare neoplasm that accounts for 0.1% to 0.2% of all malignant lung tumors.^[1]^ PMEC is characterized by squamous cells, mucus-secreting cells, and intermediate cells.^[2,3]^ Pulmonary pleomorphic adenoma (PPA) is a rare benign tumor with epithelial and myoepithelial cells and a mesenchymal component that could be chrondromyxoid stroma. PPA and PMEC are both solid tumors with pleomorphic histological appearances. The similarity of histological components on biopsy increases the difficulty of diagnosis. Here, we present the case of a rare PMEC that was distinguished from PPA by pathological examinations after three bronchoscopic biopsies and a ThinPrep cytology test (TCT). The patient benefited well from chemotherapy, and we hope that this report can provide useful evidences in the diagnosis and treatment strategy of PMEC.

## 2 Case presentation

An informed written consent was obtained from the patient for the publication of this case report and accompanying images. A 41-year-old female was admitted to our hospital with progressive cough and fever for 6 months and 10 days, respectively, in March 2017. The patient had dry cough, a maximum body temperature of 39.4 °C, no respiratory disease, and physical examination suggested no significant abnormality.

**Figure 1:**
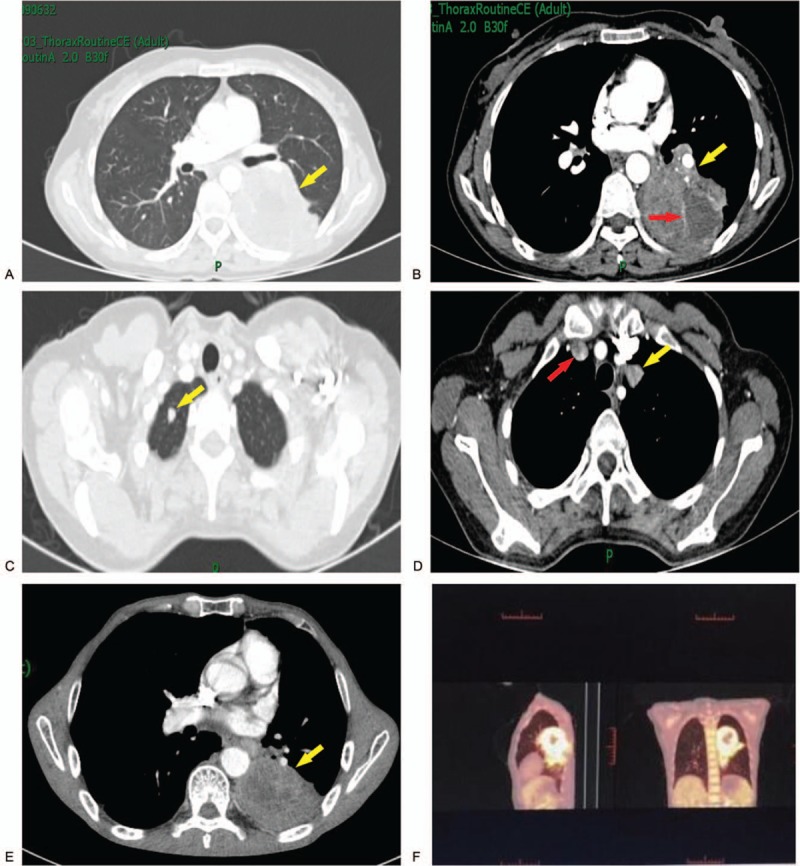
Comprehensive enhancement CT scan results. (A) A well-circumscribed soft tissue mass in the posterior segment of the left lung near the hilum (yellow arrow). (B) A low-density necrotic lesion (red arrow) in the tumor (yellow arrow) and an inhomogeneous enhancement. (C) Soft tissue in the apex of the right lung (yellow arrow). (D) Soft tissue in the left upper lobe (yellow arrow) and swollen lymph nodes in the mediastinum (red arrow). (E) The tumor size significantly shortened the posterior segment of the left lung (yellow arrow). (F) The mass near the left hilum shows high fluorodeoxyglucose (F^18^-FDG) uptake (SUVmax, 10.72).

A routine laboratory examination indicated that neuron-specific enolase (NSE), cytokeratin19 fragment antigen, and carcinoembryonic antigen 12-5 were slightly high. Contrast enhanced computed tomography (CECT) scan revealed a well-circumscribed mass of soft tissue in the posterior segment of the left lung, near the hilum, with the involvement of dorsal segment of the lower lobe. The mass was approximately 68 × 82 mm in size and connected to the pleura (Fig. 1A). A low-density necrotic lesion in the mass and inhomogeneous enhancement was observed (Fig. 1B). Soft tissue nodules (∼11 × 6 mm) in the left upper lobe and apex of the right lung, and swollen lymph nodes in the mediastinum were observed (Fig. 1C and D). In addition, the positron emission tomography computed tomography (PET--CT) scan revealed metabolic activity indicative of a mass near the left hilum, and other entities in the lung also showed high ^18^F-fluorodeoxyglucose uptake. The maximum standardized uptake value (SUVmax) of the patient was 10.72 (Fig. 1F). Therefore, the initial diagnosis upon admission was lung cancer with intrapulmonary and mediastinal metastasis.

**Figure 2:**
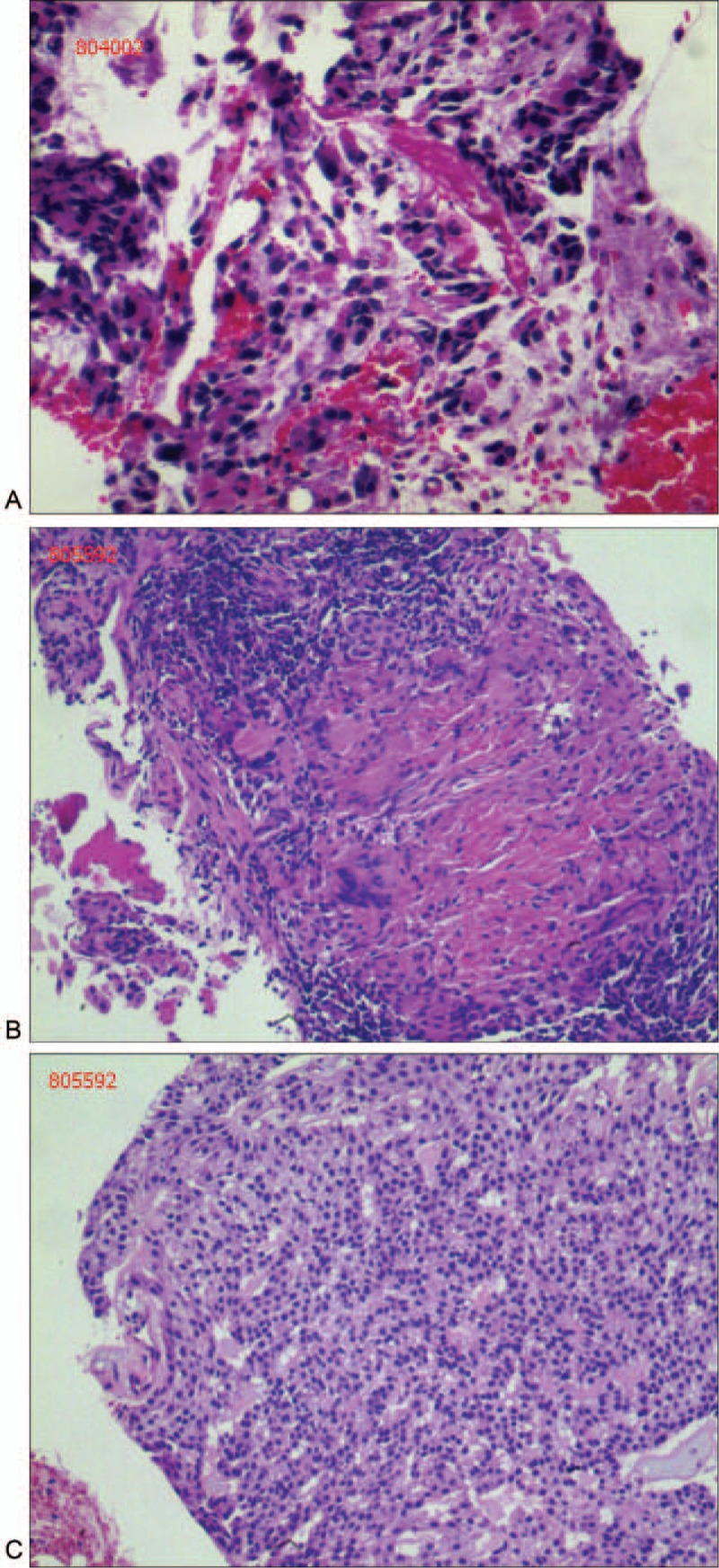
Results of the three bronchoscopy biopsies. (A) Large numbers of necrotic cells and a small number of atypical cells seen under the microscope. (B) Epithelial-like cells formed a cord-like pattern and large numbers of intracellular stroma. (C) Large numbers of epithelial-like cells.

The first bronchoscopic biopsy revealed large numbers of necrotic lesions and few atypical cells visible under the microscope (Fig. 2A). However, the results had no diagnostic value. The second bronchoscopic biopsy revealed epithelial-like cells that formed a cord-like pattern and large numbers of intracellular stroma visible under the microscope (Fig. 2B). No pathologic mitotic abnormality and mucin-secreting cells were observed. Immunohistochemistry revealed that the material was positive for cytokeratin (CK), epithelial membrane antigen (EMA), P63, cytokeratin 7 (CK7), and soluble protein-100 and negative for vimentin (VIM), cytokeratin 20 (CK20), synaptophysin (Syn), thyroidtranscription factor-1 (TTF-1), carcino-embryonic antigen (CEA), NSE, and calponin. Based on the histology and immunhistochemistry results, the second biopsy revealed a diagnosis of PPA. However, PPA is a benign pulmonary adenoma and shows no aggressive biological behavior, including malignant transformation.^[2]^ The diagnosis of PPA does not explain imaging behaviors, such as nodules in the lung and swollen lymph nodes in the mediastinum and the malignant cells that existed in the first biopsy. With the patient's positive cooperation, the third bronchoscopic biopsy was performed. Epithelial-like cells, mucus matrix, and some atypical cells of epithelial origin were observed in the third tissue specimen (Fig. 2C). Pathology revealed a low-grade variants MEC. The immunohistochemical analysis showed positive staining for P63, CK7, EMA, and cytokeratin 5/6 (CK5/6). TCT was undertaken in the third bronchoscopy, and the specimen revealed plenty of atypical cells, immunostaining was positive for CK, VIM, and EMA and negative for TTF-1, Syn, CEA, and Napsina. The third biopsies and TCT results exhibited atypical cells from the epithelium, and the biopsies were positive for MEC-specific molecular markers-P63, CK7, EMA, and CK5/6.^[4]^ Finally, the patient was diagnosed with low-grade pulmonary mucoepidermoid carcinoma (pT4N2M1).

According to the first-line chemotherapy for advanced non-small cell lung cancer (NSCLC), the patient received gemcitabine (1000 mg/m^2^ on day 1 and day 8) and carboplatin (area under the curve = 5) on day 1 every 3 weeks for 4 cycles. The patient completed 2 cycles of therapy. The mass in the left lung decreased from 68 × 22 mm to 41 × 17 mm (Fig. 1E). According to the response evaluation criteria in solid tumors (RECIST), the patient achieved partial response (PR). The patient showed no emerging recurrence or metastasis after a follow-up of 18 months. Adverse events were evaluated according to the common terminology criteria from adverse events (CTCAE), she was absence of symptoms, and the toxicity grade of the patient was grade 1.

## 3 Discussion

PMEC belongs to the salivary-gland type of lung malignant neoplasms and arises from the salivary gland-type epithelium of the central airway.^[5]^ Histologically, PMEC consists of epithelioid cells (or squamoid cells), mucin-secreting cells, and intermediate cells (or transitional cells) that are divided into high-grade and low-grade variants. These cells behave in various growth architectures, including nest, papillary, or island structures.^[6]^ Foci of calcification and ossification occurs occasionally.^[7]^ PMEC can occur at any age, and most of the patients with PMEC presented with a large bronchial obstruction with nonproductive coughs, chest pain, or post obstructive pneumonia.^[8]^ Some patients were asymptomatic, and symptoms were noted only during routine health examinations.^[9]^

PPA is a biphasic neoplasm with epithelial or myoepithelial cells and a mesenchymal component, including mucoid or chondromyxoid stroma.^[10]^ Epithelial cells and intracellular stroma were observed in patient's second biopsy. Myoepithelial participation was also reported in PMEC in 1986.^[11,12]^ The overlapping of histopathological features in the 2 mixed tumors poses a diagnostic challenge for pathologists. In our case, the second biopsy results supported the diagnosis of PPA. However, microscopic examinations of the first and third biopsies found malignant cells, CECT scan images showed intrapulmonary and mediastinum metastasis, and PET-CT revealed a high uptake tumor. Overall, these results were suggestive of a malignant tumor. The immunohistochemistry studies also proved that the tumor arised from the epithelium. According to the literature available on PPA and PMEC, hyperplasia of the epithelium in PPA might lead to the transformation of PPA into PMEC.^[2,6]^ The patient's immunohistochemistry results showed that the MEC-specific molecular markers P63, CK7, EMA, and CK5/6 were positive. We confirmed the final diagnosis of this case was PMEC not PPA.

Smetanan initially described PMEC as originating from the bronchus in 1952,^[12]^ and since then, a few cases have been described further. Besides the relative rarity, the application of bronchoscopic biopsy also increases the difficulty for pathologists to obtain an accurate diagnosis.^[13,14]^ Therefore, surgery was the first consideration for the diagnosis of PMEC. If the patients could not undergo an operation, multiple biopsies through a bronchoscope or CT-guided percutaneous were recommended. PMEC originates from the submucous myoma and are well encapsulated; and in our case, this probably explains why smear tests performed thrice showed no signs of tumor cells. However, TCT revealed plenty of atypical cells. Hence, TCT is helpful for the diagnosis of PMEC.

The routine treatment of PMEC is surgical resection. Sleeve lobectomy is frequent performed to remove the complete tumor with nodal dissection.^[9]^ There is no sufficient evidence to prove that adjuvant chemotherapy or radiotherapy is necessary for a patient with complete tumor resection.^[15]^ The CRTCl-MAML2 fusion gene generates the CRTC1-MAML2 fusion protein.^[16]^ This novel protein not only leads to up-regulation of the EGFR ligand amphiregulin but also contributes to tumor development by interfering with cell growth regulatory pathways.^[15,17,18]^ The CRTC1-MAML2 fusion gene is a possible new therapeutic target for PMEC. A few cases showed that patients with EGFR gene mutations benefit from treatment with TKI.^[19,20]^ However, patients without EGFR mutation also benefited from TKI treatments.^[21]^ Thus, the relationship between EGFR expression and TKI treatment warrants further research. However, TKI treatment was not opted for our patient because she refused genetic testing. Because of the metastasis of the malignant tumor, the patient received chemotherapy, and the regime was gemcitabine combined with carboplatin. After two cycles of chemotherapy, the mass in the left lung distinctly decreased from 68 × 22 mm to 41 × 17 mm and showed no emerging recurrence or metastasis from April 2017 to September 2018. According to the RECIST, the patient achieved PR.

Important prognostic factors of PMEC include histological typing, TNM stage, radioactivity uptake, and age.^[8]^ As opposed to high-grade PMEC, the prognosis of low-grade PMEC is excellent. Although the patient histological typing is low-grade. Unfortunately, she lost the chance of operation, based on intrapulmonary and mediastinal metastasis in the CECT scan images, the TNM stage of the patient was pT4N2M1. So, we infer our patient has poor prognosis. Besides, radioactivity uptake of PET-CT scan indicated that patients with an SUVmax > 6.5 were thought to likely have a high-uptake tumor,^[22,23]^ the SUVmax of the patient was 10.72. therefore, it was a high-uptake tumor. And high SUVmax in PET-CT had higher tumor grade, more frequent lymph node metastasis, and a worse long-term outcome.^[22]^ The prognosis is expected to be better when discovring and starting treatment early.

## 4 Conclusion

PMEC is a primary malignancy of the lung that may be microscopically disguised as PPA. In diagnostically challenging cases, multiple pathological biopsies and TCT immunohistochemical staining are necessary, besides other ancillary examinations like image studies and laboratory examination.

## Author contributions

**Formal analysis:** Jixiang Liu, Linfeng Peng.

**Investigation:** Yulan Zhong, Siyun Li.

**Methodology:** Xiangxiang Zhou, Min Zhang.

**Project administration:** Xin Gan.

**Resources:** Xiangxiang Zhou, Min Zhang.

**Software:** Xin Gan.

**Validation:** Min Zhang.

**Visualization:** Xiangxiang Zhou, Min Zhang.

**Writing – original draft:** Xiangxiang Zhou, Xingyan Yan.

**Writing – review & editing:** Xiangxiang Zhou, Min Zhang.

## References

[1] Ponea AM Marak CP Sun Y Unusual synchronous lung tumors: mucoepidermoid carcinoma and mucinous adenocarcinoma. Case Rep Oncol Med 2014;2014:183617. 24707420 10.1155/2014/183617 PMC3970349

[2] Travis WD 2015 WHO classification of the pathology and genetics of tumors of the lung. J Thorac Oncol 2015;10:S68.

[3] Westacott LS Tsikleas G Duhig E Primary epithelial-myoepithelial carcinoma of lung: a case report of a rare salivary gland type tumor. Pathology 2013;45:420–2. 23619585 10.1097/PAT.0b013e328360dfa0

[4] Zhu S Schuerch C Hunt J Review and updates of immunohistochemistry in selected salivary gland and head and neck tumors. Arch Pathol Lab Med 2015;139:55–66. 25549144 10.5858/arpa.2014-0167-RA

[5] Fink DD Lomas AM Roden AC Primary mucoepidermoid carcinoma of the lung with prominent clear cells. Proc (Bayl Univ Med Cent) 2017;30:322–4. 28670072 10.1080/08998280.2017.11929633 PMC5468030

[6] Pozgain Z Dulic G Kristek J Giant primary pleomorphic adenoma of the lung presenting as a post-traumatic pulmonary hematoma: a case report. J Thorac Cardiovasc Surg 2016;11:18. 10.1186/s13019-016-0409-z PMC4721136 26790408

[7] Roden AC Garcia JJ Wehrs RN Histopathologic, immunophenotypic and cytogenetic features of pulmonary mucoepidermoid carcinoma. Mod Pathol 2014;27:1479–88. 24743219 10.1038/modpathol.2014.72

[8] Li X Guo Z Liu J Clinicopathological characteristics and molecular analysis of primary pulmonary mucoepidermoid carcinoma: case reports and literature review. ??? 2018;9:316–23. 10.1111/1759-7714.12565 PMC5792747 29388384

[9] Xi JJ Jiang W Lu SH Primary pulmonary mucoepidermoid carcinoma: an analysis of 21 cases. World J Surg Oncol 2012;10:232. 23114230 10.1186/1477-7819-10-232 PMC3526401

[10] Moran CA Suster S Askin FB Benign and malignant salivary gland-type mixed tumors of the lung. Clinicopathologic and immunohistochemical study of eight cases. Cancer 1994;73:2481–90. 7513602 10.1002/1097-0142(19940515)73:10<2481::aid-cncr2820731006>3.0.co;2-a

[11] Nikai H el-Bardaie AM Takata T Histologic evaluation of myoepithelial participation in salivary gland tumors. Int J Oral Maxillofac Surg 1986;15:597–605. 2431078 10.1016/s0300-9785(86)80066-7

[12] Smetana HF Iverson L Swan LL Bronchogenic carcinoma; an analysis of 100 autopsy cases. Mil Surg 1952;111:335–51. 13002149

[13] Shreve P Faasse T Role of positron emission tomography-computed tomography in pulmonary neoplasms. Radiol Clin North Am 2013;51:767–79. 24010905 10.1016/j.rcl.2013.05.001

[14] Padma S Sundaram PS George S Role of positron emission tomography computed tomography in carcinoma lung evaluation. J Cancer Res Ther 2011;7:128–34. 21768697 10.4103/0973-1482.82918

[15] Chopra A Shim C Sharma N Primary salivary type lung tumor: mucoepidermoid carcinoma. Respir Med Case Rep 2013;9:18–20. 26029623 10.1016/j.rmcr.2013.03.005 PMC3949550

[16] Salem A Bell D Sepesi B Clinicopathologic and genetic features of primary bronchopulmonary mucoepidermoid carcinoma: the MD Anderson Cancer Center experience and comprehensive review of the literature. Virchows Arch 2017;470:619–26. 28343305 10.1007/s00428-017-2104-4

[17] Kumar V Soni P Garg M A comparative study of primary adenoid cystic and mucoepidermoid carcinoma of lung. Front Oncol 2018;8:153. 29868475 10.3389/fonc.2018.00153 PMC5962707

[18] Agrawal M Paul TR Uppin SG Mucoepidermoid carcinoma in carcinosarcoma of the lung - a rare combination. Turk Patoloji Derg 2016;32:122–5. 27136111 10.5146/tjpath.2014.01254

[19] Calik M Sadullahoglu C Veral A Immunohistochemical and clinicopathological analysis of primary salivary gland-type lung carcinomas. Virchows Arch 2015;467:S256.

[20] Macarenco RS Uphoff TS Gilmer HF Salivary gland-type lung carcinomas: an EGFR immunohistochemical, molecular genetic, and mutational analysis study. Modern Pathol 2008;21:1168–75. 10.1038/modpathol.2008.113 PMC2752817 18587327

[21] O’Neill ID Gefitinib as targeted therapy for mucoepidermoid carcinoma of the lung: possible significance of CRTC1-MAML2 oncogene. Lung Cancer 2009;64:129–30. 19185385 10.1016/j.lungcan.2009.01.003

[22] Park B Kim HK Choi YS Prediction of pathologic grade and prognosis in mucoepidermoid carcinoma of the lung using (1)(8)F-FDG PET/CT. Korean J Radiol 2015;16:929–35. 26175595 10.3348/kjr.2015.16.4.929 PMC4499560

[23] Krishnamurthy A Ramshankar V Majhi U Role of fluorine-18-fluorodeoxyglucose positron emission tomography-computed tomography in management of pulmonary mucoepidermoid carcinomas and review of literature. Indian J Nucl Med 2016;31:128–30. 27095092 10.4103/0972-3919.178264 PMC4815385

